# Oxidative Stress and Indicators of Brain Damage Following Pediatric Heart Surgery

**DOI:** 10.3390/antiox11030489

**Published:** 2022-02-28

**Authors:** Débora Cañizo Vázquez, Stephanie M. Hadley, Marta Pérez Ordóñez, Miriam Lopez-Abad, Anna Valls, Marta López Viñals, Bosco A. Moscoso, Sergio Benito Fernandez, Marta Camprubí-Camprubí, Joan Sanchez-de-Toledo

**Affiliations:** 1 BCNatal-Barcelona Center for Maternal Fetal and Neonatal Medicine, Hospital Sant Joan de Déu-Hospital Clinic, University of Barcelona, 08950 Barcelona, Spain; debora.canizo@sjd.es (D.C.V.); miriam.lopez.abad@gmail.com (M.L.-A.); 2 Department of Pediatrics, Boston Children’s Hospital, Boston, MA 02115, USA; stephanie.hadley@childrens.harvard.edu; 3 Pediatric Cardiology Department, Sant Joan de Déu Hospital, Cardiovascular Research Group, Sant Joan de Deu Research Institute, 08950 Barcelona, Spain; marta.perezo@sjd.es; 4 Institut de Recerca Pediàtrica, Hospital Sant Joan de Déu, 08950 Barcelona, Spain; annafrancia.valls@sjd.es; 5 Department of Anesthesia, Hospital Sant Joan de Déu, 08950 Barcelona, Spain; marta.lopezv@sjd.es; 6 Department of Cardiothoracic Surgery, Hospital Sant Joan de Déu, 08950 Barcelona, Spain; boscoalejandro.moscoso@sjd.es; 7 Department of Pediatric Critical Care, Hospital Sant Joan de Déu, Institut de Recerca Sant Joan de Déu, 08950 Barcelona, Spain; sergio.benito@sjd.es; 8 Cardiovascular Research Group, Sant Joan de Deu Research Institute, BCNatal-Barcelona Center for Maternal Fetal and Neonatal Medicine, Hospital Sant Joan de Déu-Hospital Clinic, University of Barcelona, 08950 Barcelona, Spain; 9 Department of Critical Care Medicine, University of Pittsburgh, Pittsburgh, PA 15213, USA; joan.sanchez@sjd.es

**Keywords:** 8-iso-prostaglandin F2α, pediatric cardiac surgery, oxidative stress, S100B protein

## Abstract

Pediatric cardiac surgery induces an increased oxidative stress (OS) response. Increased OS is associated with poor neurologic outcomes in neonatal populations with similar patterns of brain injury. We investigated OS and brain injury in infants undergoing heart surgery. Patients 6 months or younger, undergoing cardiac surgery with or without cardiopulmonary bypass (CPB), were included in this prospective, observational study. Patients were divided into infant (30 days–6 months) and neonatal (<30 days) groups for analysis. Urine OS biomarker 8-iso-prostaglandin F2α (8-iso-PGF2α) was quantified pre-surgery and at 0 and 24 h post-surgery. A serum brain damage biomarker S100B protein was also measured pre-surgery and at 0 and 72 h post-surgery. Amplitude-integrated electroencephalography during surgery was analyzed. Neuropsychological evaluation using the Bayley III or Vineland test was performed in all patients at 24 months of age. Sixty-two patients were included, 44 of whom underwent follow-up neurologic evaluation. 8-iso-PGF2α and S100B levels were increased after surgery. Postoperative levels of S100B were positively correlated with 8-iso-PGF2α levels 24 h after surgery (rho = 0.5224; *p* = 0.0261). There was also a correlation between immediate post-surgery levels of 8-iso-PGF2α and intra-surgery seizure burden (rho = 0.4285, *p* = 0.0205). Patients with an abnormal neurological evaluation had increased levels of S100B 72 h after surgery (*p* = 0.048). 8-iso-PGF2α levels 24 h after surgery were also related to abnormal neurologic outcomes. Levels of 8-iso-PGF2α following pediatric cardiac surgery are associated with several indicators of brain injury including brain damage biomarkers, intra-operative seizures, and abnormal neurological evaluation at follow-up, suggesting the importance of oxidative stress response in the origin of brain damage in this population.

## 1. Introduction

With recent advances in surgical techniques and critical care medicine, over 90% of pediatric patients born with congenital heart disease (CHD) survive to adulthood [1]. One of the most prevalent morbidities facing this growing population is neurocognitive impairment. Though several pre- and post-natal factors affect neurologic function, cardiac surgery during infancy has been shown to impact the development of the immature brain [2]. Pediatric heart surgery induces an overt oxidative stress (OS) response [3,4].

Free radicals normally exist in equilibrium with innate antioxidant defenses, a disruption in this equilibrium can result in damage to DNA, lipids, proteins, and cell membranes [5]. Immature cells of the oligodendroglia lineage that will later contribute to myelination are particularly susceptible to OS injury [6]. Given the higher concentrations of immature oligodendrocytes and fatty acids existing in the developing brain, infants are prone to OS-induced damage [7].

There is increasing evidence that OS increases after cardiac surgery in pediatric patients, but its association with neurologic damage and overall clinical outcomes remains uncertain [8,9]. In other vulnerable populations such as preterm infants, OS has been shown to be correlated with poor neurologic outcomes. In term neonates suffering from birth asphyxia, higher cord blood OS response is associated with the severity of the disease [10].

Furthermore, it has been reported that premature infants with higher perinatal levels of OS have poorer neurologic development at 12 months [11]. Given the similarities in brain injury pathophysiology between term infants experiencing birth asphyxia, premature newborns, and babies born with CHD, we hypothesized that OS may play a role in neurologic dysfunction around cardiac surgery [12,13]. In this prospective study, we sought to characterize the relationship between urinary free 8-iso-prostaglandin F2α (8-iso-PGF2α), a biomarker of OS, and clinical and neurological outcomes in infants undergoing cardiac surgery.

## 2. Materials and Methods

### 2.1. Patients

Infants of six months of age or younger that require cardiac surgery, with or without cardiopulmonary bypass (CPB), were included in this prospective, observational study. Patients were divided into neonatal (<30 days at time of surgery) and infant (30 days–6 months) groups for analysis. Those with a known genetic syndrome with neurological implications and history of birth asphyxia were excluded. Clinical data including length of stay (LOS), days of mechanical ventilation, pre-surgical treatments, intensive care unit (ICU), and overall hospital LOS were recorded. REDCap was used to store electronic data [14]. Parents or legal guardians of all included patients signed a written informed consent. This study was reviewed and approved by the local institution’s Ethics Committee (PIC 120-17) and was conducted in accordance with the Declaration of Helsinki. 

### 2.2. Surgical Management

Anesthetic management was performed following institutional cardiac anesthesia protocols which included inhaled isoflurane, opioids (fentanyl), and muscle relaxants. No benzodiazepines nor barbiturates were used. The perfusion strategy, in those patients undergoing CPB, involved pulsatile full flow at 100–150 mL per minute to achieve an optimal arterial pressure. The target temperature during CPB was 22–34 °C. Some selected surgeries required periods of deep hypothermic circulatory arrest. Continuous hemofiltration was used in all patients during CPB. Standard early therapy included milrinone combined with dopamine and epinephrine if needed. Postoperative analgesia and sedation were managed with continuous infusions of morphine (10–40 mg/kg/h) and dexmedetomidine (0.3–0.7 mcg/kg/min).

### 2.3. Oxidative Stress Biomarkers 

Urine samples were collected before surgery and at 0 and 24 h post-surgery. Samples were stored at −80 °C until analysis. Free urine concentrations of 8-iso-PGF2α, were quantified using an enzyme-linked immunoassay (Cell BioLabs, Inc., San Diego, CA, USA) [15]. Levels were adjusted for urinary creatinine excretion and expressed in ng/mg of creatinine (ng/mg Cr).

### 2.4. Serum Brain Damage Biomarkers

Blood samples were drawn immediately before surgery and at 0, 24, and 72 h post-surgery. Samples were drawn from indwelling venous or arterial catheters and stored at −80 °C until analysis. S100B protein levels were quantified using enzyme-linked immunoassays (Liaison S100B DET, Palex Medical, Barcelona, Spain). 

### 2.5. Amplitude-Integrated Electroencephalogram

Brain electrical activity during surgery was monitored with a continuous amplitude-integrated electroencephalogram (aEEG) monitor (NicoletOne^TM^, Natus, Middleton, WI, USA). The decision was made to utilize the aEEG device as opposed to a traditional EEG given its relative diagnostic simplicity, allowing for personnel outside neurology to interpret the tracings. This device recorded a 2-channel electroencephalogram from 4 central and parietal hydrogel electrodes corresponding to C3, C4, P3, and P4, respectively. Impedance below 20 Ω was assured. All aEEG records were analyzed by two blinded neonatologists in accordance with Hellström-Westas classifications [16]. Continuous and discontinuous background patterns were considered normal and electrical seizures were identified. In those patients with intra-operative seizures, the total seizure burden was calculated in minutes.

### 2.6. Neurologic Evaluation

Infants were evaluated at 24 months of age. Neuropsychological testing, including Bayley-III cognitive, language, and motor evaluations, was performed by one trained psychologist blinded to the clinical details of the children. Most of the included patients were evaluated using The Spanish version of the Bayley Scales of Infant and Toddler Development^®^, Third Edition (Bayley-III^®^) [17].

The COVID-19 pandemic impacted the ability to perform in-person evaluations over a period of 6 months. During this time, telematic interviews using the Vineland evaluation set were performed as a substitute neurologic evaluation. The Vineland test is a standardized norm-referenced assessment tool used to measure adaptative behavior of infants from birth to age 90 focusing on the following domains: communication, daily living skills, socialization, and motor skills [18].

All the evaluated patients were classified into two groups: *Normal outcome (NO)*, defined as patients with a Bayley score over 85, and those with a Vineland score over 86, or *Abnormal Outcome (AO)*. 

### 2.7. Statistical Analysis

Normality and homogeneity were analyzed for all the variables. Mean and standard deviation or mean and interquartile range were used for main demographic descriptions. All of them were compared using the Student’s *t*-test or the Mann–Whitney U test based on distribution. Continuous variables were analyzed using the Kruskal–Wallace test. Spearman correlation coefficient was used for correlations. Confounding factors were analyzed using linear regression. Logistic regression was performed to obtain the prediction model. Statistical analyses were performed using SPSS version 25 (IBM, Armonk, NY, USA) and STATA v13 package. Statistical significance was considered when *p* < 0.05.

## 3. Results

### 3.1. Demographic Charactiristics of Our Population

**Figure 1:**
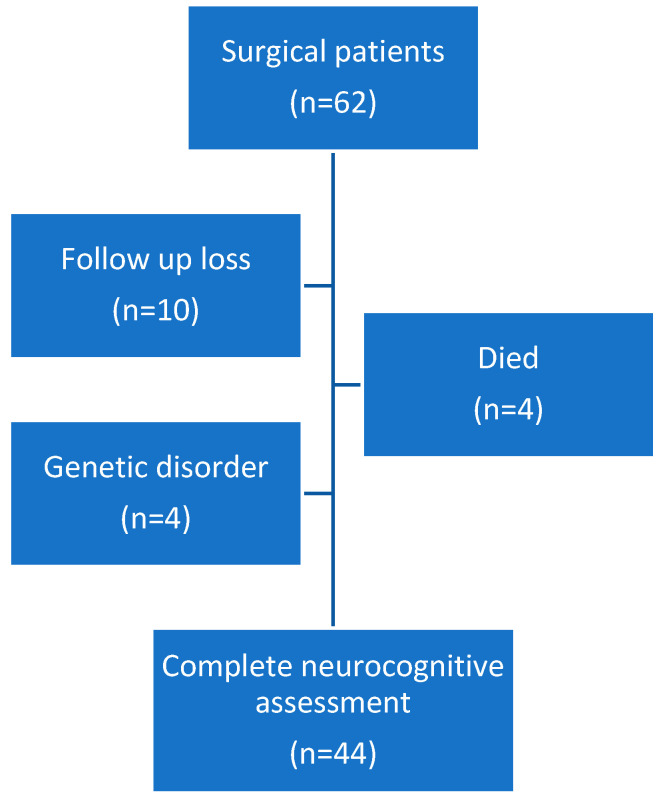
Flow chart of patients.

Sixty-two patients were included. All of them underwent cardiac surgery between November 2017 and February 2019. Patients were divided into neonatal (<30 days at time of surgery) and infant (30 days–6 months) groups for analysis. All infant patients underwent CPB surgery (*n* = 25). In neonatal patients, some of them require CPB surgery (*n* = 12) and some non-CPB (*n* = 25). Forty-four underwent neurologic evaluation at 2 years of age. Four patients died, two of them in the post-operative period and the other two at home due to concurrent processes. Four patients were excluded from the study due to genetic disorders. Ten patients were lost to follow-up (Figure 1). The median age at the time of surgery was 21 days [IQR 8–96]. Patient demographics are displayed in Table 1.

Table 2 includes a description of the different types of congenital heart defects included in the sample.

### 3.2. Urine Oxidative Stress Biomarkers

**Figure 2:**
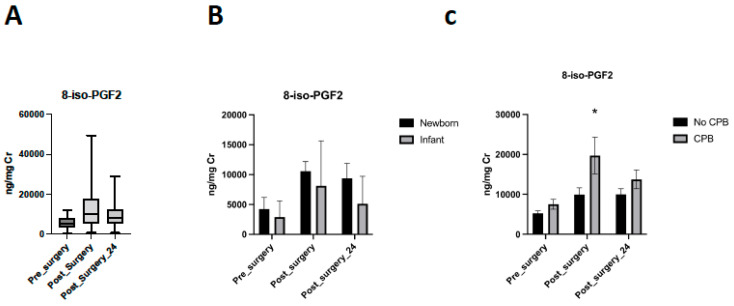
Levels of 8-iso-PGF2α. (**A**) Evolution of 8-iso-PGF2α in the perioperative period. (**B**) Levels of 8-iso-PGF2α in the perioperative period considering age group. (**C**) Evolution of 8-iso-PGF2α in the perioperative period considering the requirement of cardio-pulmonary bypass (CPB) during the surgery (* *p* = 0.0214).

Levels after surgery were higher than baseline levels (11.26 [7.4–13.7] ng/mg vs. Cr 5.3 [3.9–7.3] ng/mg Cr; *p* = 0.002) and similarly levels at 24 h post-operatively (9.6 [6.1–11.2] ng/mg Cr) were higher than baseline (*p* = 0.012) (Figure 2A).

Neonatal patients presented higher levels of 8-iso-PGF2α immediately post-operatively, but in both groups, perioperative differences persist (*p* = 0.013; *p* = 0.0004) (Figure 2B). Neonatal patients presented a decreased rate of 8-iso-PGF2α clearance after surgery (*p* = 0.032).

Considering the use of cardiopulmonary bypass during the surgery, no differences were detected in pre-surgical levels between patients with CPB or without CPB (*p* = 0.4853). All infants require CPB surgery. CPB patients had increased levels than non-CPB patients immediately post-surgery (*p* = 0.0214). These differences were not observed 24 h after surgery (*p* = 0.9724) (Figure 2C). A regression analysis was performed to correct 8-iso-PGF2α levels after surgery for age as a possible confounder without differences (*p* = 0.3831).

None of the main patient characteristics, clinical conditions or pre-surgical treatments, seem to have had an influence on pre-surgical levels of 8-iso-PGF2α (*p* > 0.05). No difference in 8-iso-PGF2α levels, considering the use of pre-surgical prostaglandin infusion, were detected pre-surgical: *p* = 0.7758; post-surgical: *p* = 0.3407; post-surgical24: *p* = 0.7067).

In those patients with electrical seizures during surgery, there was a correlation between seizure burden and immediate post-surgery levels of 8-iso-PGF2α (rho = 0.4285, *p* = 0.0205).

### 3.3. Brain Damage Biomarkers

**Figure 3:**
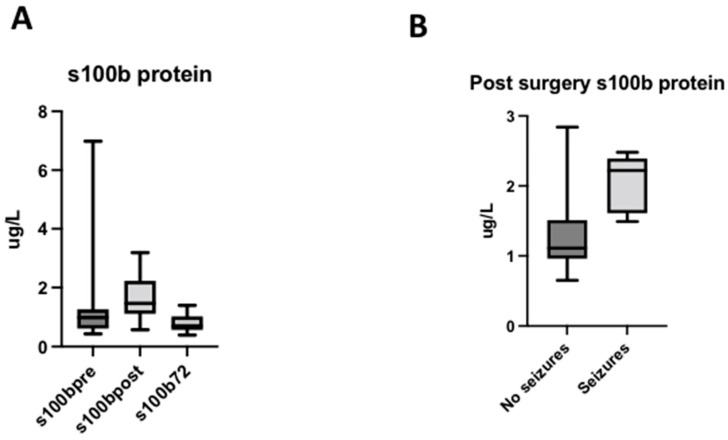
Levels of S100B protein. (**A**) Evolution of S100B protein in the perioperative period. (**B**) Levels of S100B protein depending on the presence of electrical seizures during the surgery.

S100B levels significantly increased after surgery with an immediate postoperative peak (PRE: 0.85 [0.65–1.09]; POST: 1.49 [1.32–1.65] (*p* = 0.003). At 72 h after surgery, S100B decreased to lower levels than those measured at baseline (0.66 [0.61–0.79] (*p* = 0.001) (Figure 3A). The postoperative peak of S100B was higher in newborns (1.68 [1.38–2.226]) than in infant patients (1,24 [1.03–1.52]) (*p* = 0.0011). No significant differences were found based on the use of CBP (*p* = 0.5200). 

Patients with electrical seizures during the surgery had increased levels of S100B in the post-operative period (*p* = 0.018) (Figure 3B).

**Figure 4:**
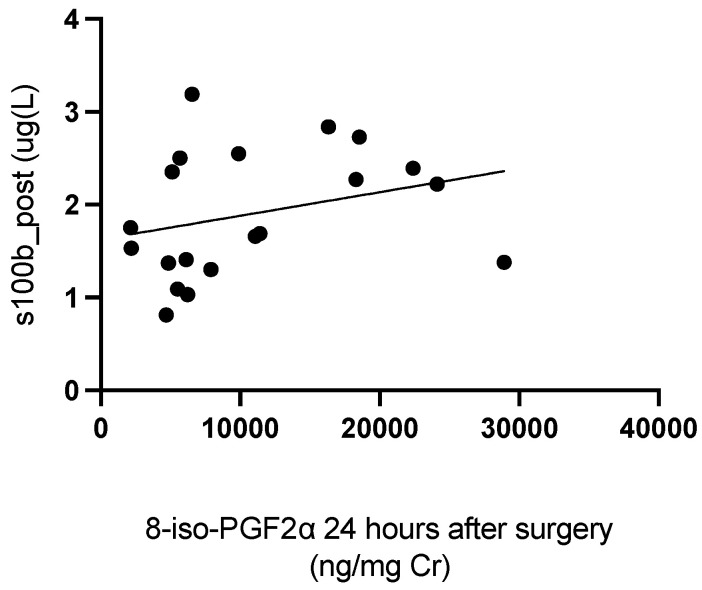
Correlation between levels of S100B immediately post-surgery and levels of 8-iso-PGF2α 24 h after surgery.

In neonatal patients, postoperative levels of S100B were positively correlated with levels of 8-iso-PGF2α 24 h after surgery (rho = 0.5224; *p* = 0.0261) (Figure 4).

### 3.4. Neurologic Evaluation

Of the 62 infants enrolled, 44 (66.6%) were evaluated for neurodevelopmental outcomes at a mean age (±SD) of 24.4 ± 0.9 months (Figure 1). The results of the neurocognitive tests were analyzed considering prematurity, but no differences were found between groups (*p* = 0.5607). Twenty-five patients were evaluated using the Bayley-III test, 34 using the Vineland test, and 15 patients were evaluated using both tests. The following tables (Table 3 and Table 4) summarize the Bayley and Vineland results.

Thirteen patients (29.55%) were included in the AO group. All these patients were operated on during the neonatal period (younger than 30 days).

**Figure 5:**
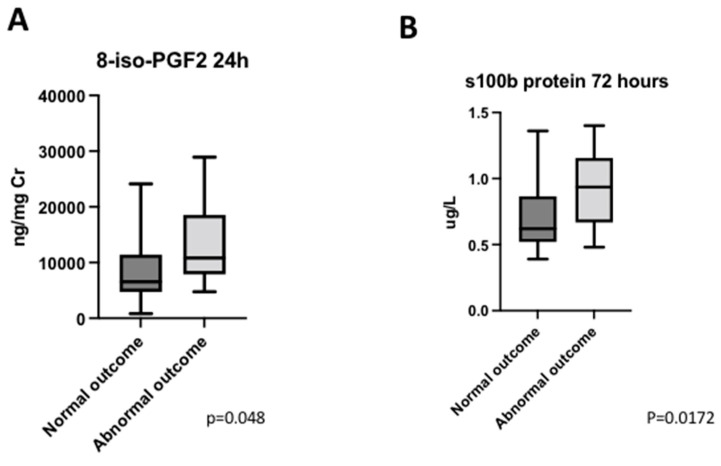
Neurological outcome. (**A**) Distribution of 8-iso-PGF2 levels 24 h after the surgery considering neurological outcome. (**B**) Distribution of S100B protein levels 72 h after the surgery considering neurological outcome.

Pre-surgical and immediate post-surgical levels of 8-iso-PGF2α were similar between NO and AO groups (*p* = 0.2205; *p* = 0.5592); but at 24 h, those patients with AO had higher levels (13.5 [6.2–2.0] vs. 6.2 [4.9–10.8], *p* = 0.048) (Figure 5A).

Presurgical and immediate postoperative levels of S100B were similar between both groups (*p* = 0.1620; *p* = 0.7441). At 72 h post-surgery, S100B levels were higher in patients with worse neurologic outcomes (*p* = 0.0172) (Figure 5B).

**Figure 6:**
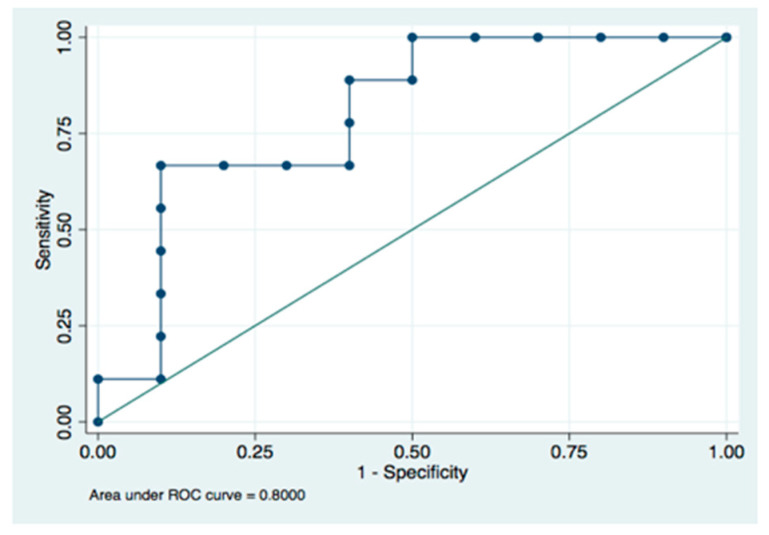
ROC curve for levels of S100B at 72 h.

Peri-surgical biological parameters (S100B and 8-iso-PGF2α) were used to create a predictive model for abnormal neurological outcomes at two years. In neonatal patients, levels of S100B at 72 h were a strong indicator of patients with an increased risk of AO, (AUC of 0.800) (Figure 6). 8-iso-PGF2α levels 24 h post-surgery, when analyzed alone, did not have enough power to predict AO (AUC = 0.7188). Levels of S100B at 72 h analyzed together with 8-iso-PGF2α levels at 24 h post-surgery increased the AUC to 0.822, but it was not statistically different compared to the predictions made utilizing levels of S100B at 72 h after surgery alone (*p* = 0.447).

## 4. Discussion

We have shown that the OS response, quantified by urinary 8-iso-PGF2α, in infants undergoing cardiac surgery is correlated with serum biomarkers of brain damage, intra-operative electrical seizures, and abnormal neurological outcomes. All these findings point in the same direction, suggesting a possible relation between brain damage following pediatric cardiac surgery and OS. 

8-iso-PGF2α is the product of nonenzymic, free radical-catalyzed peroxidation of arachidonic acids. It has been widely used as a biomarker of lipid peroxidation, being one of the most reliable indicators of OS [15]. In our population, levels of 8-iso-PGF2α immediately post-surgery were increased compared to patient baseline. The pathophysiology of OS during cardiac surgery, particularly involving CPB, is multi-factorial [19,20,21]. In addition, intra-operative changes in blood pressure and perfusion may cause ischemia-reperfusion injury, a process that can be significant in both CPB and non-CPB surgeries [22]. In a previous study, time of extracorporeal circulation and levels of malondialdehyde were related [23]. Our group has recently demonstrated that lipid peroxidation is increased in pediatric patients undergoing cardiac surgery, especially in neonatal patients, who had higher and more prolonged elevations of 8-iso-PGF2α following the procedure [4]. The smaller size of the patients in relation to the bypass circuit and the immaturity of neonatal antioxidant defense systems may be part of the explanation [24].

Our study identified a strong correlation between OS and a previously described brain damage biomarker S100B protein. The S100B protein is a calcium-mediated protein secreted by astrocytes and it has been linked to clinical neurologic outcomes in many pediatric conditions [25,26]. Increases in S100B protein levels after cardiac surgery in adults have been correlated with many neurological symptoms. In 2017, Trakas and colleagues described that neuronal-specific enolase and S100B levels increased after CPB, returning to baseline levels by postoperative day 7. They also described a correlation between levels of S100B and circulatory arrest time [27]. Similarly, Bar-Yoseff and colleagues, in a prospective study, identify postoperative S100B levels as an early marker for brain injury in those children undergoing cardiac surgery [28].

Intra-operative electrical seizures were related to significantly higher post-operative OS. Furthermore, the total intra-operative seizure burden was correlated with post-operative 8-iso-PGF2α. In this line, animal studies have shown increased lipid peroxidation after seizure onset [29]. Damage from lipid peroxidation is one of the main contributing factors to white matter injury, which is a principal contributor to long-term neuronal injury in patients with CHD [30]. Moreover, perioperative seizures have been previously reported to be common in this patient population, though we did not find any clear relationship with 2-year neurodevelopmental outcomes. Other electrical alterations such as delayed recovery in electroencephalographic background activity have been associated with increased risk of early mortality and worse neurodevelopment [31].

In our population, around 30% of the patients requiring cardiac surgery during the first month of life had an abnormal neurological evaluation at two years. These data are similar to previously published findings, despite heterogeneity in methodology between the studies. Motor and cognitive delay in the first 2–3 years, lower intelligence quotient (IQ), decreased performance in executive function, language, and fine motor and visual–motor skills at school age, with increased rates of psychosocial maladjustment and educational needs, were some of the main issues in this population [32,33].

Early diagnosis of neurological alterations is difficult and any clinical biomarker to detect such injuries would represent a significant advancement. Various serologic markers of brain injury have been identified, but to our knowledge, this is the first time that oxidative stress response to cardiac surgery has been demonstrated to play a role in impaired neurologic development. Urine levels of 8-iso-PGF2α 24 h after surgery and S100B protein levels at 72 h after surgery were increased in patients with AO; both biomarkers are strong tools to promptly identify patients at higher risk of poor neurologic outcomes.

Our study had several limitations. Firstly, the small size of the sample could have had an effect on the statistical power. Moreover, we only examined one biomarker of OS. Despite 8-iso-PGF2α being generally accepted as one of the most stable in vivo biomarkers of OS, there are other OS pathways that could also play a role in brain injury. In addition, although we have demonstrated preliminary correlations between OS and AO, it will be important to correlate these alterations with neuroimaging findings and continued long-term neurologic evaluations. A longer follow-up period will also improve the quality of our results.

## 5. Conclusions

In conclusion, infants undergoing cardiac surgery experience significant post-operative OS response measured by 8-iso-PGF2α. Increased OS partially results from hypoxic-reoxygenation injury and changes in cerebral blood flow, which affect not only CPB patients but also neonates undergoing non-CPB procedures. We have shown that increased 8-iso-PGF2α levels are associated with increased S100B levels, a previously identified brain damage biomarker, as well as with intra-operative electrographic seizures and poor neurologic outcomes at 2 years of life. Future studies encompassing a larger number of patients should investigate the role of OS response as an early marker of brain injury following pediatric cardiac surgery.

## Figures and Tables

**Table 1:** Patients’ characteristics (only those with neurological evaluation).

Patient Characteristics	Newborns (*n* = 29)	Infant Patients (*n* = 15)	*p*-Value
Male sex	62.16%	52%	0.426
Prematurity (<37 weeks)	10.81%	20%	0.314
Gestational age (weeks)	39.03(1.8)	38.02(2.8)	0.11
Birth weight (kg)	3.14 (0.56)	2.7 (0.78)	0.021
Presurgical prostaglandin infusion	88.89%	31.82%	0.0001
Sub-atmospheric therapy	27.78%	4.5%	0.017
Cardiopulmonary bypass	32.43%	100%	0.0001
Stat category 1 2 3 4 5	32.43 37.84 8.11 18.92 2.179	44 28 20 8 0	0.296
Biventricular repair	78.38%	88%	0.321
Left ventricle outflow tract obstruction	45.95%	16%	0.012
Hospital length of stay (days)	36.97 (8.8)	14.9 (5)	0.066
Electric seizures during surgery	27.03% (10)	5.4% (2)	0.156
Seizure burden (min)	18.68 (8.2)	3.75 (2.6)	0.2605

**Table 2:** Patient diagnoses.

Type of Defect	Number of Patients
Coarctation of the aorta	11
Transposition of the great arteries	8
Ventricular septal defect	6
Tetralogy of Fallot	7
Pulmonary atresia	4
Double outlet right ventricle	2
Truncus arteriosus	2
Tricuspid atresia	1
Total anomalous pulmonary venous return	2
Others	1

**Table 3:** Neurodevelopmental outcomes using the Bayley scales of infant and toddler development (Bayley-III).

Domain	Score
**Cognitive score** ≥85 70–84 <70	90 (85–100) 72% 20% 8%
**Language score** ≥85 70–84 <70	89 (79.41–93.68) 64% 28% 8%
**Perceptive language**	9 (8–10)
**Expressive language**	7 (6–9.8)
**Motor score** ≥85 70–84 <70	100 (88–102) 80% 16% 4%
**Fine motor skills**	11 (9–12)
**Gross motor skills**	8 (7–9)

**Table 4:** Neurodevelopmental outcomes using the Vineland test.

Domain	Score
**Communication** ≥85 70–84 <70	67.65% 26.47% 5.88%
**Daily living skills** ≥85 70–84 <70	85.29% 11.76% 2.94%
**Socialization** ≥85 70–84 <70	58.82% 35.29% 5.88%
**Motor skills** ≥85 70–84 <70	70.59% 29.41% 0%

## Data Availability

Data is contained within the article.
